# Depressive symptoms and its influencing factors of older people with cataracts in China: a national cross-sectional survey

**DOI:** 10.3389/fpubh.2025.1504275

**Published:** 2025-01-29

**Authors:** Tengfei Niu, Shiwei Cao, Xiaoyu Wang, Xiaobing Xian, Chunyang Luo, Jingxi Ma

**Affiliations:** ^1^Department of Basic Courses, Chongqing Medical and Pharmaceutical College, Chongqing, China; ^2^The Second Clinical College, Chongqing Medical University, Chongqing, China; ^3^College of Paediatrics, Chongqing Medical University, Chongqing, China; ^4^Thirteenth People’s Hospital of Chongqing, Chongqing, China; ^5^Department of Neurology, Chongqing General Hospital, Chongqing University, Chongqing, China

**Keywords:** cataracts, depressive symptoms, older people, CLHLS, random forest

## Abstract

**Background:**

Depressive symptoms are a common complication in patients with cataracts and may exacerbate cataract symptoms. Therefore, it is important to focus on depressive symptoms and their influencing factors in older people with cataracts. The purpose of this study was to investigate the prevalence rate of depressive symptoms and influencing factors in Chinese older people with cataracts.

**Methods:**

Descriptive analyses were used to report the sociodemographic characteristics, lifestyle, health status, and depressive symptoms of old people with cataracts in China. The chi-square test was used to compare differences between subjects with different demographic characteristics. Binary logistic regression was used to analyze the factors that influenced the depressive symptoms of cataract patients. Meanwhile, a random forest model was developed in this study to rank the importance of the influencing factors.

**Results:**

Three hundred and six (25.27%) of 1,211 cataract patients included in this study suffered from depressive symptoms. Logistic regression analysis suggested that poor economic situation (AOR = 3.162, 95%CI: 1.719–5.817), social participation (AOR = 1.530, 95%CI: 1.053–2.222), having hearing disorder (AOR = 1.445, 95%CI: 1.040–2.008), poor self-reported health status (AOR = 2.646, 95%CI: 1.705–4.106), poor life satisfaction (AOR = 3.586, 95%CI: 1.652–7.784) were risk factors for depressive symptoms in cataract patients and consumption of fresh fruits (AOR = 0.587, 95%CI: 0.369–0.933) was a protective factor for depressive symptoms in cataract patients. The results of the random forest showed that self-reported health status was the most important factor influencing depressive symptoms in cataract patients. The other factors, in order of importance, were life satisfaction, economic situation, fruits, hearing disorder, and social participation.

**Conclusion:**

The results suggested that the development of depressive symptoms in cataract patients was influenced by various factors. Medical staff should monitor these influencing factors more closely when treating and caring for patients with cataracts.

## 1 Introduction

The ocular condition known as cataracts is characterized by a pathological clouding of the lens and a clinical manifestation of vision loss, and its incidence increases significantly with aging (1). Cataract is the primary cause of blindness and the second most common cause of visual impairment worldwide (2). It was considered that over 15 million people worldwide were blind due to cataracts in 2020, accounting for about 45% of the 33.6 million blindness cases all over the world (3). Besides, it was estimated that the prevalence rate of cataracts in China will reach 33.34% among people aged 45–89 years, and the total of cataract cases would reach 240.83 million by the end of 2050 (2). Cataract-induced vision loss not only influences socioeconomic levels and access to education but also reduces patients’ life quality and increases their risk of developing psychological disorders such as depression (4, 5).

Depressive symptoms are a common complication in patients with ocular diseases (5). Studies have reported a combined prevalence rate of depression and depressive symptoms of up to 25% in cataract patients (6). The high prevalence rate of depressive symptoms in patients with cataracts may be due to age-related lens changes, which decrease the transmission of short-wave light, disrupting circadian rhythms and leading to sadness and sleeplessness (7). Secondly, fear of blindness, heavy financial burden, limitation of physical activity, difficulty in social interactions, and decline in life quality may all cause depressive symptoms in cataract patients (8–10). In addition, patients with depressive symptoms are more likely to show HPA axis disorders (11), leading to increased levels of glucocorticoids in the body. Exposure to high levels of glucocorticoids induces changes in the expression of relevant genes and activation of receptors in the lens epithelium (12), exacerbating the symptoms of cataracts. Thus, the high prevalence rate of depressive symptoms in cataract patients and the detrimental effect of depressive symptoms on cataracts create a vicious circle.

Even though depressive symptoms are common among ophthalmic patients in clinical practice, depression often goes unrecognized or untreated in ophthalmology clinics or ophthalmology outpatient clinics (13). Given that symptomatic interventions can improve depressive symptoms in visually impaired older people (14), it’s crucial to focus on the main influencing factors that lead to the development of depressive symptoms in cataract patients. Previous studies have investigated the prevalence rate of depressive symptoms and influencing factors in cataract patients in Chinese populations (8, 15, 16). However, by recruiting small and convenient samples, they did not consider the national representativeness of the influencing factors in Chinese older people. In addition, the research methods in previous related studies tended to be traditional logistic regression analysis. As a machine learning algorithm, Random Forest (RF) has become an excellent research tool due to its powerful classification ability and easy-to-interpret learning mechanism. In recent years, RF has been widely used in the medical aspect for diagnosis and classification of diseases (17), prediction of clinical outcomes (18), and estimation of the significance of exposure to disease-causing factors (19). In summary, we aimed to use the Chinese Longitudinal Healthy Longevity Survey (CLHLS) database to analyze the factors influencing the depressive symptoms of cataract patients in older people in terms of three dimensions: socio-demographic characteristics, lifestyle, and health status, and to apply the RF to the importance ranking.

## 2 Materials and methods

### 2.1 Data source

CLHLS began in 1998 and aimed to conduct a nationally representative survey of Chinese people aged over 65. The survey covers 23 provinces, municipalities, and autonomous regions across China, involving about 85% of the population. It collects information on the basic characteristics, socioeconomic characteristics, behavioral habits, dietary situation, and physical health of older people (20). The CLHLS received ethical approval from the Biomedical Ethics Committee of Peking University in China (IRB00001052-13074). Each participant completed an informed consent form before data collection.

### 2.2 Sample

**Figure 1:**
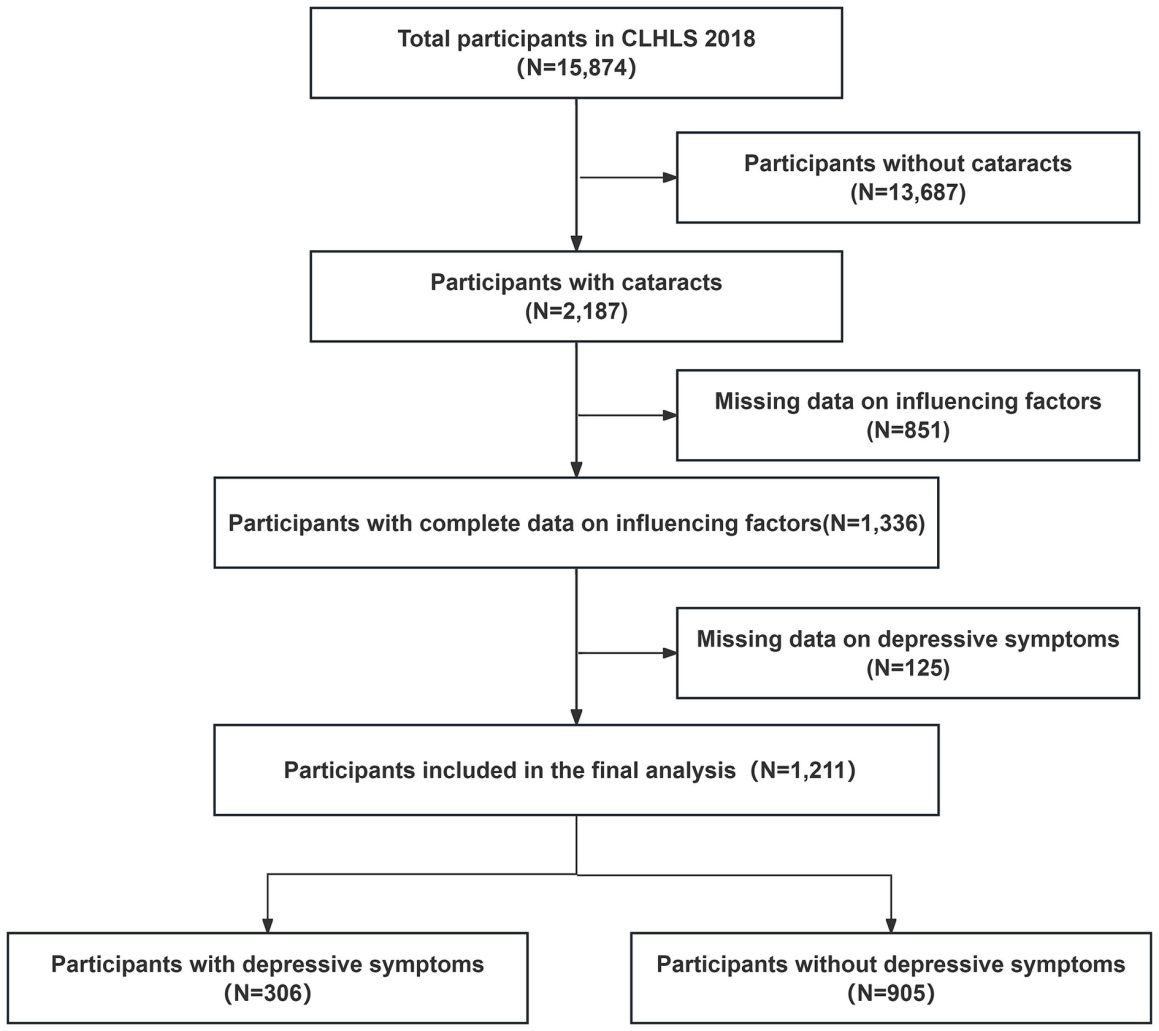
Flow chart of the study sample.

The study sample selection was based on the question in the questionnaire “Have you been diagnosed with cataracts?” Respondents who answered “yes” were involved in our study. A total of 15,874 participants were surveyed for the 2018 CLHLS, of which 2,187 participants were diagnosed with cataracts. After subsequent exclusion of missing data on depressive symptoms and possible influencing factor variables, 1,211 participants aged 65 years and older were included in the analysis. The detailed selection process is shown in Figure 1.

### 2.3 Patient characteristics

Based on previous studies about depressive symptoms (21–24), we screened 27 possible influencing factors from three dimensions: sociodemographic characteristics, lifestyle, and health status. The detailed variable assignments are in Supplementary Table S1.

Sociodemographic characteristics included age, gender, marital status, education, ethnic group, living arrangements, economic situation, and residence.

Lifestyle included fruits, vegetables, cooking oil, dietary tastes, sleep time, smoking, drinking, drinking boiled water, manual labor, and social participation.

Health status included Body Mass Index (BMI), abdominal obesity, hearing disorder, hypertension, diabetes, heart disease, self-reported health status, physical dysfunction and life satisfaction.

### 2.4 Assessment of depressive symptoms

The 10-item Center for Epidemiological Studies Depression scale (CESD-10) has been used to assess depressive symptoms in older people. Studies have verified that the scale is reliable for Chinese older people (25). Ten items are included in the scale, and the options for each item include (1) never or hardly (1 day); (2) seldom (1–2 days); (3) sometimes or often (3–4 days); and (4) usually or always (5–7 days). These four options were assigned values of 0, 1, 2, and 3. “Are you hopeful for the future?” and “Do you feel as happy as you did when you were younger?” were reverse-assigned before being summed. Total scores ranged from 0 to 30. Individuals with a depression score of 10 or greater were defined as having depressive symptoms (26).

### 2.5 Statistical analysis

Categorical variables were described by frequencies and percentages. The Chi-square test was used to compare the differences between different characteristics in the presence or absence of depressive symptoms. To further explore the factors influencing depressive symptoms in cataract patients, we used binary logistic regression models for analysis. The results of the regression analyses were reported as *β*, standard error, Wald’s chi-square, *p*-value, AOR, and 95% confidence interval (CI). To assess the importance of different influencing factors, we performed an RF importance ranking of the variables with statistical significance in the logistic regression model. As a machine-learning method based on the principles of ensemble learning, RF can handle classification and regression analysis, and its prediction results are accurate with stable predictive ability. Additionally, it can provide a simple visualization method to accurately rank the importance of relevant factors (27). Compared to traditional regression methods, the RF algorithm can avoid increasing estimated parameters or being insensitive to outliers when dealing with multi-level categorical variables, demonstrating strong resistance to interference. Therefore, applying RF can reduce bias, tolerate outliers, and decrease overfitting, thereby achieving more accurate and stable results (28). The samples were randomly divided into training and test sets on a 7:3 basis, the best parameters of the model were obtained through grid optimization, and the model was tested by a 10-fold cross-validation method. The variables were ranked in terms of importance based on the average reduction in the Gini index. Specifically, the greater the average decrease in the Gini index of a variable, the more important the variable is (29). Sensitivity, specificity, recall, accuracy, F1 score, and area under the curve (AUC) were used to evaluate the performance of the random forest model. All data analyses were performed using SPSS 26.0 and R 4.3.0, and *p* < 0.05 (two-sided) was considered statistically significant.

## 3 Results

### 3.1 Sociodemographic characteristics and depressive symptoms in Chinese cataract patients

Table 1 shows the descriptive results of sociodemographic characteristics and their chi-square test results with depressive symptoms. Three hundred and six (25.27%) of the 1,211 patients suffered from depressive symptoms, 67.13% of the patients were aged ≥80 years, 44.43% were male, 42.28% were married, 38.23% were illiterate, 30.64% received primary school education or below, and 31.13% received secondary education or above. Most patients were of Han nationality (94.38%), 79.27% lived with their families, and 8.51% had poor economic situation. Patients living in rural areas accounted for 68.46% of the total sample.

**Table 1:** Sociodemographic characteristics and depressive symptoms in Chinese cataract patients.

Variables	Total (*n* = 1,211)	Normal (*n* = 905)	Depressive symptoms (*n* = 306)	Statistic	*p*
Age, *n*(%)				χ^2^ = 4.21	**0.040**
65–79	398 (32.87)	312 (34.48)	86 (28.10)		
≥80	813 (67.13)	593 (65.52)	220 (71.90)		
Gender, *n*(%)				χ^2^ = 2.12	0.145
Female	673 (55.57)	492 (54.36)	181 (59.15)		
Male	538 (44.43)	413 (45.64)	125 (40.85)		
Marital status, *n*(%)				χ^2^ = 9.79	**0.002**
Unmarried	699 (57.72)	499 (55.14)	200 (65.36)		
Married	512 (42.28)	406 (44.86)	106 (34.64)		
Education, *n*(%)				χ^2^ = 10.67	**0.005**
Illiteracy	463 (38.23)	322 (35.58)	141 (46.08)		
Primary school or below	371 (30.64)	289 (31.93)	82 (26.80)		
Secondary school or above	377 (31.13)	294 (32.49)	83 (27.12)		
Ethnic group, *n*(%)				χ^2^ = 2.79	0.095
Others	68 (5.62)	45 (4.97)	23 (7.52)		
Han	1,143 (94.38)	860 (95.03)	283 (92.48)		
Living arrangements, *n*(%)				χ^2^ = 13.19	**0.001**
Living with family	960 (79.27)	739 (81.66)	221 (72.22)		
Living alone	188 (15.52)	127 (14.03)	61 (19.93)		
Living in an institution	63 (5.20)	39 (4.31)	24 (7.84)		
Economic situation, *n*(%)				χ^2^ = 61.14	**<0.001**
Good	302 (24.94)	260 (28.73)	42 (13.73)		
Common	806 (66.56)	596 (65.86)	210 (68.63)		
Poor	103 (8.51)	49 (5.41)	54 (17.65)		
Residence, *n*(%)				χ^2^ = 0.24	0.621
Rural	829 (68.46)	623 (68.84)	206 (67.32)		
Urban	382 (31.54)	282 (31.16)	100 (32.68)		

χ^2^, Chi-square test.

The chi-square test results showed that age, marital status, education, living arrangements, and economic situation had an apparent effect on the depressive symptoms in cataract patients (Table 1). Specifically, cataract patients who were ≥ 80 years (71.90% vs. 65.52%, *p* = 0.040), unmarried (65.36% vs. 55.14%, *p* = 0.002), and illiterate (46.08% vs. 35.58%, *p* = 0.005) were more likely to experience depressive symptoms. Those living with family (72.22% vs. 81.66%, *p* = 0.001) and having good economic situation (13.73% vs. 28.73%, *p* < 0.001) were less likely to have depressive symptoms.

### 3.2 Lifestyle and depressive symptoms of Chinese cataract patients

The basic information on lifestyle characteristics and the results of their chi-square test with depressive symptoms are shown in Table 2. The proportion of cataract patients who ate fruits and vegetables every day was 32.20 and 70.02%, respectively. Most patients preferred to consume vegetable oil (94.72%) and drink boiled water (98.27%), while 31.96% had a bland dietary taste. The highest number of patients (538) had a sleep time between 7 and 9 h per day at night. About 29.07% of the patients had a habit of smoking, 22.96% had a habit of drinking, 65.32% used to engage in manual labor regularly, and 33.61% engaged in social participation.

**Table 2:** Lifestyle and depressive symptoms in Chinese cataract patients.

Variables	Total (*n* = 1,211)	Normal (*n* = 905)	Depressive symptoms (*n* = 306)	Statistic	*p*
Fruits, *n*(%)				χ^2^ = 26.48	**<0.001**
No	218 (18.00)	136 (15.03)	82 (26.80)		
Sometimes	603 (49.79)	452 (49.94)	151 (49.35)		
Often	390 (32.20)	317 (35.03)	73 (23.86)		
Vegetables, *n*(%)				χ^2^ = 17.43	**<0.001**
No	30 (2.48)	17 (1.88)	13 (4.25)		
Sometimes	333 (27.50)	227 (25.08)	106 (34.64)		
Often	848 (70.02)	661 (73.04)	187 (61.11)		
Cooking oil, *n*(%)				χ^2^ = 0.06	0.807
Animal oil	64 (5.28)	47 (5.19)	17 (5.56)		
Vegetable oil	1,147 (94.72)	858 (94.81)	289 (94.44)		
Dietary tastes, *n*(%)				χ^2^ = 0.21	0.649
Bland	387 (31.96)	286 (31.60)	101 (33.01)		
Non-bland	824 (68.04)	619 (68.40)	205 (66.99)		
Sleep time, *n*(%)				χ^2^ = 6.83	**0.033**
< 7 h	452 (37.32)	319 (35.25)	133 (43.46)		
7-9 h	538 (44.43)	418 (46.19)	120 (39.22)		
> 9 h	221 (18.25)	168 (18.56)	53 (17.32)		
Smoking, *n*(%)				χ^2^ = 3.03	0.082
No	859 (70.93)	630 (69.61)	229 (74.84)		
Yes	352 (29.07)	275 (30.39)	77 (25.16)		
Drinking, *n*(%)				χ^2^ = 0.35	0.555
No	933 (77.04)	701 (77.46)	232 (75.82)		
Yes	278 (22.96)	204 (22.54)	74 (24.18)		
Drinking boiled water, *n*(%)				χ^2^ = 0.123	0.725
No	21 (1.73)	15 (1.66)	6 (2.00)		
Yes	1,190 (98.27)	890 (98.34)	300 (98.00)		
Manual labor, *n*(%)				χ^2^ = 0.33	0.566
No	420 (34.68)	318 (35.14)	102 (33.33)		
Yes	791 (65.32)	587 (64.86)	204 (66.67)		
Social participation, *n*(%)				χ^2^ = 23.79	**<0.001**
No	804 (66.39)	566 (62.54)	238 (77.78)		
Yes	407 (33.61)	339 (37.46)	68 (22.22)		

χ^2^, Chi-square test.

### 3.3 Health status and depressive symptoms of Chinese cataract patients

Table 3 shows the descriptive results of the health status of cataract patients and the results of the chi-square test with depressive symptoms. About 12.47% of the patients had a BMI < 18.5, while 8.34% had a BMI > 28, and nearly half (47.89%) of the patients had abdominal obesity. Hearing disorder, hypertension, diabetes, and heart disease were present in 39.55, 63.25, 32.95, and 42.36% of the cataract patients, respectively. Approximately 22.13% had impaired daily activity ability. In terms of self-reported health status, 44.76% of the patients considered it good, 37.49% considered it common and 17.75% considered it poor. Only 3.72% of patients were dissatisfied with their life quality status.

**Table 3:** Health status and depressive symptoms in Chinese cataract patients.

Variables	Total (*n* = 1,211)	Normal (*n* = 905)	Depressive symptoms (*n* = 306)	Statistic	*P*
BMI, *n*(%)				χ^2^ = 8.55	**0.036**
< 18.5	151 (12.47)	100 (11.05)	51 (16.67)		
18.5–24	599 (49.46)	460 (50.83)	139 (45.42)		
24–28	360 (29.73)	274 (30.28)	86 (28.10)		
≥ 28	101 (8.34)	71 (7.85)	30 (9.80)		
Abdominal obesity, *n*(%)				χ^2^ = 0.75	0.385
No	631 (52.11)	465 (51.38)	166 (54.25)		
Yes	580 (47.89)	440 (48.62)	140 (45.75)		
Hearing disorder, *n*(%)				χ^2^ = 14.30	**<0.001**
No	732 (60.45)	575 (63.54)	157 (51.31)		
Yes	479 (39.55)	330 (36.46)	149 (48.69)		
Hypertension, *n*(%)				χ^2^ = 0.00	0.951
No	445 (36.75)	333 (36.80)	112 (36.60)		
Yes	766 (63.25)	572 (63.20)	194 (63.40)		
Diabetes, *n*(%)				χ^2^ = 3.25	0.071
No	812 (67.05)	594 (65.64)	218 (71.24)		
Yes	399 (32.95)	311 (34.36)	88 (28.76)		
Heart disease, *n*(%)				χ^2^ = 0.10	0.751
No	698 (57.64)	524 (57.90)	174 (56.86)		
Yes	513 (42.36)	381 (42.10)	132 (43.14)		
Physical dysfunction, *n*(%)				χ^2^ = 18.89	**<0.001**
No	943 (77.87)	732 (80.88)	211 (68.95)		
Yes	268 (22.13)	173 (19.12)	95 (31.05)		
Self-reported health status, *n*(%)				χ^2^ = 88.88	**<0.001**
Good	542 (44.76)	465 (51.38)	77 (25.16)		
Common	454 (37.49)	325 (35.91)	129 (42.16)		
Poor	215 (17.75)	115 (12.71)	100 (32.68)		
Life satisfaction, *n*(%)				χ^2^ = 105.99	**<0.001**
Good	854 (70.52)	703 (77.68)	151 (49.35)		
Common	312 (25.76)	188 (20.77)	124 (40.52)		
Poor	45 (3.72)	14 (1.55)	31 (10.13)		

χ^2^, Chi-square test; BMI, Body Mass Index.

Patients who were thin (16.67% vs. 11.05%, *p* = 0.036) and overweight (9.80% vs. 7.85%, *p* = 0.036), had hearing disorder (48.69% vs. 36.46%, *p* < 0.001), and had physical dysfunction (31.05% vs. 19.12%, *p* < 0.001) were more likely to have depressive symptoms. Cataract patients with depressive symptoms had poor self-reported health status (32.68% vs. 12.71%, *p* < 0.001) and poor life satisfaction (10.13% vs. 1.55%, *p* < 0.001).

### 3.4 Logistic regression analysis of depressive symptoms among Chinese cataract patients

Logistic regression analysis suggested that economic situation, fruit consumption, social participation, hearing disorder, self-reported health status, and life satisfaction were independent factors influencing depressive symptoms in cataract patients (Table 4). Cataract patients with poor economic situation were more likely to develop depressive symptoms than those with good economic situation (AOR = 3.162, 95%CI: 1.719–5.817). Consumption of fresh fruits was a protective factor for depressive symptoms in cataract patients, with cataract patients who ate fresh fruits almost every day being 41.3% less likely to develop depressive symptoms compared to those who did not eat fruits (95%CI: 0.369–0.933). Patients with social participation tended to develop depressive symptoms compared to those without social participation (AOR = 1.530, 95%CI: 1.053–2.222). Patients with hearing disorders were more likely to show depressive symptoms than those without (AOR = 1.445, 95%: 1.040–2.008). Moreover, cataract patients with poor self-reported health status were 2.646 times more likely to exhibit depressive symptoms than those with good self-reported health status (95%CI: 1.705–4.106). Cataract patients with poor life satisfaction were 3.586 times more likely to exhibit depressive symptoms than those with good life satisfaction (95%CI: 1.652–7.784).

**Table 4:** Multivariate analysis of depressive symptoms in Chinese cataract patients.

Variables	B value	Wald value	AOR (95%CI)	*P*
Age, *n*(%)				
65–79			1.00	
≥ 80	−0.066	0.108	0.936 (0.632–1.387)	0.742
Gender, *n*(%)				
Female			1.00	
Male	−0.051	0.064	0.950 (0.641–1.409)	0.800
Marital status, *n*(%)				
Unmarried			1.00	
Married	−0.190	0.928	0.827 (0.563–1.217)	0.335
Education, *n*(%)				
Illiteracy			1.00	
Primary school or below	−0.240	1.483	0.787 (0.535–1.157)	0.223
Above Primary school	0.014	0.004	1.014 (0.643–1.599)	0.952
Ethnic group, *n*(%)				
Others			1.00	
Han	−0.561	3.318	0.571 (0.312–1.044)	0.069
Living arrangements, *n*(%)				
Living with family			1.00	
Living alone	0.280	1.608	1.323 (0.858–2.040)	0.205
Living in an institution	0.593	3.289	1.810 (0.953–3.436)	0.070
Economic situation, *n*(%)				
Good			1.00	
Common	0.509	5.961	1.663 (1.105–2.501)	0.015*
Poor	1.151	13.698	3.162 (1.719–5.817)	<0.001*
Residence, *n*(%)				
Rural			1.00	
Urban	−0.037	0.044	0.964 (0.682–1.361)	0.834
Fruits, *n*(%)				
No			1.00	
Sometimes	−0.450	5.313	0.638 (0.435–0.935)	0.021*
Often	−0.533	5.074	0.587 (0.369–0.933)	0.024*
Vegetables, *n*(%)				
No			1.00	
Sometimes	−0.390	0.734	0.677 (0.277–1.653)	0.392
Often	−0.784	3.055	0.457 (0.190–1.100)	0.081
Cooking oil, *n*(%)				
Animal oil			1.00	
Vegetable oil	0.274	0.637	1.315 (0.672–2.573)	0.425
Dietary tastes, *n*(%)				
Bland			1.00	
Non-bland	0.019	0.014	1.019 (0.743–1.399)	0.907
Sleep time, *n*(%)				
< 7 h			1.00	
7-9 h	−0.048	0.080	0.954 (0.686–1.325)	0.777
> 9 h	−0.005	0.000	0.995 (0.643–1.539)	0.982
Smoking, *n*(%)				
No			1.00	
Yes	−0.259	1.570	0.772 (0.515–1.157)	0.210
Drinking, *n*(%)				
No			1.00	
Yes	0.336	2.785	1.399 (0.943–2.076)	0.095
Drinking boiled water, *n*(%)				
No			1.00	
Yes	0.539	0.639	1.714 (0.457–6.421)	0.424
Manual labor, *n*(%)				
No			1.00	
Yes	−0.038	0.044	0.963 (0.676–1.371)	0.833
Social participation, *n*(%)				
No			1.00	
Yes	0.425	4.975	1.530 (1.053–2.222)	0.026*
BMI, *n*(%)				
< 18.5			1.00	
18.5–24	−0.387	2.801	0.679 (0.432–1.068)	0.094
24–28	−0.240	0.769	0.787 (0.460–1.345)	0.381
≥ 28	0.216	0.385	1.241 (0.628–2.452)	0.535
Abdominal obesity, *n*(%)				
No			1.00	
Yes	0.042	0.057	1.043 (0.740–1.468)	0.811
Hearing disorder, *n*(%)				
No			1.00	
Yes	0.368	4.811	1.445 (1.040–2.008)	0.028*
Hypertension, *n*(%)				
No			1.00	
Yes	0.081	0.222	1.084 (0.774–1.519)	0.638
Diabetes, *n*(%)				
No			1.00	
Yes	−0.263	2.002	0.769 (0.535–1.106)	0.157
Heart disease, *n*(%)				
No			1.00	
Yes	0.054	0.099	1.056 (0.753–1.479)	0.753
Physical dysfunction, *n*(%)				
No			1.00	
Yes	0.255	1.651	1.291 (0.874–1.096)	0.199
Self-reported health status, *n*(%)				
Good			1.00	
Common	0.631	12.138	1.879 (1.318–2.680)	<0.001*
Poor	0.973	18.814	2.646 (1.705–4.106)	<0.001*
Life satisfaction, *n*(%)				
Good			1.00	
Common	0.787	20.323	2.196 (1.560–3.091)	<0.001*
Poor	1.277	10.431	3.586 (1.652–7.784)	0.001*

*p-value < 0.05. B value, Regression Coefficient; Wald value, Wald Chi-Squared Statistic; AOR, Adjusted Odds Ratio; CI, Confidence Interval; BMI, Body Mass Index.

### 3.5 Random Forest results

**Figure 2:**
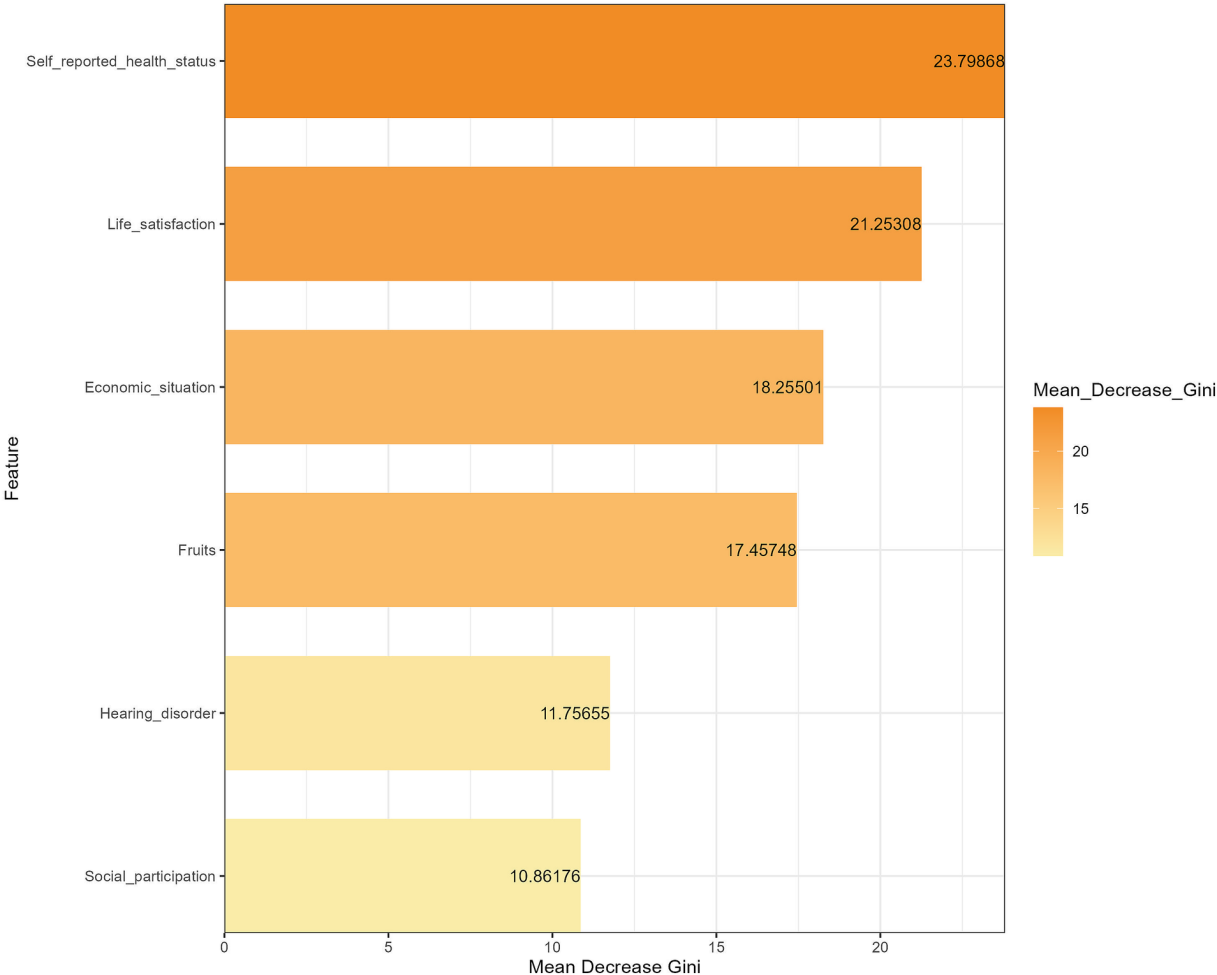
Ranking of the importance of different factors on depressive symptoms among Chinese cataract patients.

In order to assess the importance of significant influencing factors in the logistic regression model on the impact of depressive symptoms, we further established an RF model to rank the variable importance. Specifically, this study established a random forest model with the parameter ‘mtry’ set to 3 and the parameter ‘ntree’ set to 500 by randomly selecting 70% of the overall data as the training set and 30% as the test set. The RF algorithm measured the impact of each variable on the dependent variable through the importance score of the variables. The greater the average decrease in the Gini index, the more important the variable. The results of RF indicated that self-reported health status was the key factor affecting depressive symptoms in cataract patients, with other factors influencing depressive symptoms in cataract patients in the following order of importance: life satisfaction, economic situation, fruits, hearing disorder, and social participation (Figure 2). Table 5 shows the performance metrics of the random forest model on the training and test sets, all of which indicate that the model performs well.

**Table 5:** Model performance evaluation metrics.

	AUC	Accuracy	Recall	Specificity	F1 Score	Sensitivity
Training set	0.800	0.824	0.948	0.838	0.890	0.948
Test set	0.697	0.732	0.875	0.790	0.830	0.875

AUC, Area Under Curve.

## 4 Discussion

To our knowledge, this study is the first to report on the prevalence and influencing factors of depressive symptoms in older people with cataracts in China. Based on a nationally representative database, we explored the factors that influenced depressive symptoms in cataract patients from three dimensions: sociodemographic characteristics, lifestyle, and health status. We also performed a random forest importance ranking of the significant factors. We observed that depressive symptoms prevalence rate in Chinese older people with cataracts was 25.27%, which is comparable to the results of Liu et al. (16). Dandan Zhang et al. reported a prevalence rate of 18.00% of depressive symptoms in Chinese cataract patients, compared with 5.2% in healthy individuals (8), which suggests that cataract patients have higher risk of developing depressive symptoms. Moreover, a 16-year longitudinal survey confirmed the higher risk of clinically diagnosed depression in cataract patients (30). In addition, several studies in the United States, Australia, and Japan have consistently indicated that cataract patients experience more severe depressive symptoms than healthy individuals (31–33).

Individuals with cataracts in poor economic situations are at increased risk of developing depressive symptoms compared to those in better economic situations. A meta-analysis carried out by Lorant et al. indicated that individuals of low socioeconomic status were at higher risk of depression, with each 1% increase in income rank being associated with a 0.74 decline in the log odds of developing depressive symptoms (34). In general, cataract patients with poor economic situations have poor life quality and daily health care and may have more irregularities in cataract intervention care (35), which may exacerbate cataracts to some extent. And the deterioration of the disease and the low quality of life will increase the psychological burden of patients and increase the risk of depressive symptoms. In addition, people with poor economic situations may not have sufficient social support resources to deal with their mental health problems, leading to an increased risk of depressive symptoms in these patients.

Fruits and vegetables have been treated as a crucial part of a healthy diet all the time, and their rich nutrients (e.g., polyphenols, flavonoids, and antioxidant vitamins) are able to counteract oxidative stress and inflammation, playing an important role in improving cataract progression (36). Also, the protective effect of fruits and vegetables against depressive symptoms has long been demonstrated (37). Their beneficial effects on depression may be attributed to the high levels of antioxidants, dietary fiber, and vitamins they contain that play a role in inflammation, oxidative stress, and gut microbiota (38). Interestingly, only fruits were observed to have a protective effect on depressive symptoms in our study, which may be due to differences in how fruits and vegetables are consumed. In China, vegetables are usually cooked, leading to the degradation or loss of certain beneficial nutrients, such as the antioxidant vitamin C, whereas fruits are usually consumed raw. This nutrient loss during cooking may lead to the unobserved protective effect of vegetables on depressive symptoms in cataract patients in our study. Further research is needed to find the effects of different vegetable processing methods on depressive symptoms.

Studies have shown that social participation is a crucial mediator in mitigating the effects of visual impairment on depressive symptoms (39). The study by Simone Croezen et al. also indicated that social participation can reduce the risk of depressive symptoms in older people, which contradicts our research findings (40). Activity Theory suggests that engaging in social activities may help maintain a positive self-perception and boost self-esteem, thereby buffering the negative impact of aging on mental health (41). The Role Accumulation Theory holds that a large number of role identifications imply extensive social support, which can help older people maintain a certain level of social participation, thereby promoting positive mental health outcomes (42). Social participation may provide opportunities for older people to establish social relationships and engage in emotional exchanges, resulting in higher levels of perceived connection and lower levels of loneliness (43). However, our study showed that cataract patients who had social participation were more likely to have depressive symptoms. We hypothesized that due to severe vision problems, older people with cataracts feel isolated and stressed and have difficulty adapting to social participation, leading to an inability to engage in effective social participation (44). In addition, comparisons with others, difficulties with self-expression, and stigma and discrimination may exacerbate feelings of shame and lower self-esteem in people with cataracts, and this negative change in self-image may also lead to depressive symptoms (45). Once people experience negative social events or social rejection, they may become inertial or resistant to social participation, which may become a vicious cycle (46) and cause high depressive symptoms prevalence.

The prevalence rate of hearing disorder is higher in cataract patients, and hearing disorder has a significant correlation with depressive symptoms in cataract patients. The prevalence rate of poor hearing and vision is high in older people, which is considered to be an age-related degeneration (47). A cohort study showed that patients with both hearing and visual disorders were at significantly higher risk of developing depressive symptoms, with patients who have dual sensory impairment showing more severe depressive symptoms (48). Loneliness and social isolation caused by dual sensory deprivation in cataract patients with hearing disorders may contribute to their high prevalence of depressive symptoms (49, 50).

Our findings showed that poor self-reported health status and poor life satisfaction were significantly associated with depressive symptoms in cataract patients. Research has indicated that older people with visual impairment are more likely to report their poor health (51). Patients with cataracts may have certain lifestyle and health management challenges, such as an unbalanced diet and insufficient physical activity, which may raise the risk of developing chronic diseases and lead to a greater likelihood of reporting poor health. Persistent physical discomfort, dysfunction, and decreased life quality after multiple chronic conditions can increase the risk of developing depressive symptoms (52, 53). Life satisfaction refers to an individual’s overall well-being, which is strongly linked to health outcomes such as pain, physical functioning, chronic disease, and mortality (54). When people experience vision loss, well-being may be significantly reduced and accompanied by a range of adverse health conditions, resulting in a greater likelihood of reporting poor life satisfaction. And previous studies have also reported that people who report poor life satisfaction are more likely to experience depressive symptoms (55), which explains the significant association between life satisfaction and depressive symptoms in cataract patients.

The results of this study using RF indicate that self-reported health status is the most important factor affecting depressive symptoms in cataract patients, followed by life satisfaction, economic situation, fruits, hearing disorder, and social participation. Therefore, strategies to improve depressive symptoms in older people with cataracts should primarily focus on enhancing their awareness and ability to self-evaluate their health. Public health departments urgently need to take action by widely disseminating health knowledge through diverse channels such as community forums, television broadcasts, and online platforms to deepen cataract patients’ understanding and assessment of their own health conditions. Specifically, for this vulnerable group, educational forms that are more aligned with their needs should be adopted, such as face-to-face lectures and home visit services, to overcome their reading barriers with written materials. Public health education and promotion should emphasize the importance of individuals assessing and perceiving their own health conditions, encouraging cataract patients to regularly self-evaluate their health and promptly identify and report psychological issues. At the same time, regular health check-ups and chronic disease screenings should be promoted as routine practices, with systematic follow-up management for diagnosed patients, assisting them in comprehensively understanding their health conditions through effective communication. Additionally, this study calls for the government and all sectors of society to pay attention to the economic difficulties faced by older people with cataracts and to alleviate their financial burden through improved medical insurance policies and medical assistance. In terms of nutritional guidance, a balanced diet should be advocated, especially increasing the intake of foods rich in vitamins and minerals, particularly fruits, to enhance the body’s resistance. For cataract patients with hearing disorder, hearing screenings should be implemented, and hearing aids or interventions should be provided in a timely manner to reduce the negative impact of hearing impairments on mental health. Comprehensive measures should be taken from multiple dimensions, such as medical care, lifestyle, mental health, and social support, to improve the life satisfaction and well-being of older people with cataracts. It is worth noting that the complex relationship between social participation and depressive symptoms in older people with cataracts still needs further exploration and research. In summary, public health departments should formulate and implement comprehensive intervention strategies to comprehensively improve the depressive symptoms of older people with cataracts.

## 5 Summary

Based on the CLHLS database, this study evaluated the prevalence of depressive symptoms among older people with cataracts aged 65 and above in China and identified related influencing factors. A random forest model was used to rank the importance of these factors. The results showed that self-reported health status is the most important factor influencing depressive symptoms in cataract patients. Other factors were ranked in order of importance as life satisfaction, economic situation, fruits, hearing disorder, and social participation. Based on this, medical staff should monitor these influencing factors more closely when treating and caring for patients with cataracts.

## 6 Limitations

There are several limitations of this study. Firstly, the selection of influencing factors for this study was limited by the structure of the questionnaire. Therefore, we cannot guarantee that all potential factors were included in this study. Second, the variables we studied were all measured by self-reported questions, which may lead to recall bias. Third, the data we used were obtained from a cross-sectional survey, which precludes any causal inference about the relationship between depressive symptoms and their influencing factors in cataract patients.

## Data Availability

Publicly available datasets were analyzed in this study. This data can be found at: the data of CLHLS are available at: https://opendata.pku.edu.cn/dataverse/CHADS.
