# Supplementary material for: Depressive symptoms and its influencing factors of older people with cataracts in China: a national cross-sectional survey
Source: Front Public Health. 2025 Jan 29;13:1504275. doi: 10.3389/fpubh.2025.1504275 (PMC11813796; doi:10.3389/fpubh.2025.1504275)
Supplement: Supplementary file 1 [file Table_1.DOCX]

Supplementary Material

# Supplementary Table 1. Questionnaire Items and Classification Criteria of Covariates.

| **Variables** | **Questionnaire Items** | **Classification and assignment of variables** |
| --- | --- | --- |
| Gender | What’s your gender? | 0=Female  1=Male |
| Age | What’s your current age? | 0=65-79  1=≥80 |
| Marital status | What’s your current marital status？ | 0=Unmarried  1=Married |
| Education | What’s your education level? | 0=Illiteracy  1=Primary school or below  2=Above primary school |
| Ethnic group | What’s your ethnic group? | 0=Others  1=Han |
| Living arrangements | What’s your living arrangements? | 0=Living with family  1=Living alone  2=Living in institution |
| Economic situation | How do you rate your economic status compared with others in your local area? | 0=Good  1=Common  2=Poor |
| Residence | What’s your current residence of interviewee？ | 0=Rural  1=Urban |
| Fruits | Do you eat fresh fruit? | 0=No  1=Eat  2=Almost every day |
| Vegetables | Do you eat fresh vegetables? | 0=No  1=Eat  2=Almost every day |
| Cooking oil | What kind of grease do you mainly use for cooking? | 0=Animal oil  1=Vegetable oil |
| Dietary tastes | What kind of flavor do you mainly have? | 0=Bland  1=Non-bland |
| Sleep time | How many hours do you sleep normally? | 0= <7h  1= 7-9h  2= >9h |
| Smoking | Do you smoke at the present time? Did you smoke at the past? | 0=No  1=Yes |
| Drinking | Do you drink alcohol at the present time? Did you drink alcohol in the past? | 0=No  1=Yes |
| Drinking boiled water | What kind of water do you usually drink? | 0=No  1=Yes |
| Manual labor | Have you done physical labor regularly? | 0=No  1=Yes |
| Social participation | Do you now perform Social activities(organized)? | 0=No  1=Yes |
| BMI | What’s your BMI? | 0= <18.5  1=18.5-24  2=24-28  3= ≥28 |
| Abdominal obesity | Do you have abdominal obesity？ | 0=No  1=Yes |
| Hearing disorder | Do you have any difficulty with your hearing? | 0=No  1=Yes |
| Hypertension | Are you suffering from hypertension ? | 0=No  1=Yes |
| Diabetes | Are you suffering from diabetes? | 0=No  1=Yes |
| Heart disease | Are you suffering from heart disease ? | 0=No  1=Yes |
| Physical dysfunction | Do you have physical dysfunction？ | 0=No  1=Yes |
| Self-reported health status | How do you rate your health at present? | 0=Good  1=Common  2=Poor |
| Life satisfaction | How do you rate your life at present? | 0=Good  1=Common  2=Poor |

**Note:**BMI:Body Mass Index
